# Systematic Echocardiographic Assessment of the Univentricular Heart Across the Stages of Fontan Palliation: A Practical Guide with Focus on Emerging 3D and 4D Imaging Modalities and Thromboembolic Complications from AEPC Imaging Working Group

**DOI:** 10.3390/jcm15093520

**Published:** 2026-05-05

**Authors:** Massimiliano Cantinotti, Pietro Marchese, Giovanni Di Salvo, Nadia Assanta, Guglielmo Capponi, Inga Voges, Francesca Raimondi, Almudena Ortiz Garrido, Sylvia Krupickova, Giulia Pasqualin, Heynric Grotenhuis, Martin Köestenberger, Beatrice Bonello, Owen Miller, Colin J. McMahon

**Affiliations:** 1 Fondazione CNR-Regione Toscana G. Monasterio (FTGM), 54100 Massa, Italy; 2 Paediatric Cardiology and Congenital Heart Disease, Woman and Children’s Health Department, University of Padua, Experimental Cardiology, Paediatric Research Institute (IRP), 35122 Padua, Italy; 3 Department for Congenital Cardiology and Pediatric Cardiology, University Hospital Schleswig-Holstein, Campus Kiel, 24105 Kiel, Germany; 4 Division of Pediatric Cardiology, Ospedali Riuniti, ASST Azienda Ospedaliera Papa Giovanni XXIII, 24127 Bergamo, Italy; 5 Section of Pediatric Cardiology, Hospital Materno Infantil of Malaga, 29011 Málaga, Andalucia, Spain; 6 Royal Brompton and Harefield NHS Foundation Trust, London SW3 6NP, UK; 7 Pediatric Cardiology and Adult Congenital Disease Heart Centre, IRCCS Policlinico San Donato, San Donato Milanese, 20097 Milan, Italy; 8 Department Pediatric Cardiology, University Medical Center Groningen, 9713 GZ Groningen, The Netherlands; 9 Department of Pediatrics, Division of Pediatric Cardiology, Medical University Gratz, 8036 Graz, Austria; 10 Great Ormond Street Hospital for Children, London WC1N 3JH, UK; 11 Department Pediatric Cardiology, Evelina London Children’s Hospital, London SE1 7EH, UK; 12 Department Paediatric Cardiology, Children’s Health Ireland at Crumlin, D12 N512 Dublin, Ireland

**Keywords:** echocardiography, univentricular heart, fontan, thrombus, clot

## Abstract

Although echocardiography remains the primary imaging modality for assessing Fontan palliation, a standardized systematic approach for evaluating the univentricular heart throughout the different stages of palliation has yet to be established. This document aims to provide a narrative review and practical guide for the echocardiographic assessment of the univentricular heart at various phases of Fontan palliation. Additional objectives include highlighting the potential of advanced three-dimensional (3D) and four-dimensional (4D) imaging modalities, as well as outlining a systematic strategy for detecting thromboembolic complications. We propose a sequential framework for echocardiographic evaluation, encompassing key anatomical and functional components of the univentricular heart. This includes the atrial septum and pulmonary veins, ventricular function, atrioventricular valve, aorta and neo-aorta, sub-aortic region, aortic arch, and pulmonary arteries. Furthermore, we detail the assessment of pulmonary blood supply at different stages of palliation, including the modified Blalock–Taussig–Thomas (mBTT) shunt, Sano conduit, Glenn procedure, and total cavo-pulmonary connection (TCPC). A comprehensive analysis of potential thrombus formation sites is provided, along with diagnostic pitfalls. Additionally, we outline methods for screening for extracardiac clots following Glenn and TCPC procedures. This document serves as a practical guide for the systematic echocardiographic evaluation of the univentricular heart across all stages of Fontan palliation, offering guidance for clinical practice. It also explores the capabilities of emerging 3D and 4D echocardiographic techniques in univentricular heart assessment and provides an in-depth review of thromboembolic complications, emphasizing key diagnostic challenges.

## 1. Background

Echocardiography is the primary modality for the assessment of the univentricular heart across different stages of univentricular palliation. Despite the presence of robust literature [1,2,3,4,5,6,7,8,9,10,11,12,13,14,15,16], the standards and recommendations for a systematic approach for the evaluation of univentricular heart in the different phases of univentricular palliation has not yet been established. Furthermore, the additional diagnostic and prognostic values of newer echo modalities, e.g., speckle tracking echocardiography (STE) [17,18,19,20,21,22,23,24,25,26,27,28,29,30,31,32,33,34,35,36,37,38,39,40,41,42,43,44,45] and three-dimensional (3D) echocardiography [9,10,11,25,31] in the assessment of univentricular heart, which is further supported by multiple studies. Despite this, the application of new echo modalities into clinical practice remains limited. Thromboembolic complications are a well-known complication across all the stages of univentricular palliation [46,47,48,49,50,51,52,53,54]. A systematic assessment of all potential sites of thrombus formation has not yet been standardized. Extracardiac clots are also common after superior cavo-pulmonary connection (Glenn or HemiFontan) and total cavo-pulmonary connection (TCPC) surgery, though their routine assessment is still limited [55,56].

The aim of the present document is to provide a narrative review and practical guide for the systematic echocardiographic assessment of the univentricular heart at different stages of Fontan palliation. The other aims are (i) to focus on the potentiality of newer 3D and 4D modalities in the evaluation and follow-up of univentricular heart through different phases of Fontan palliation; and (ii) to provide a systematic approach for the search of thromboembolic complications.

## 2. Methods

A comprehensive literature review was conducted in January 2026 using three major medical databases: the National Library of Medicine (PubMed/MEDLINE), ScienceDirect, and the Cochrane Library. The search strategy included both Medical Subject Headings (MeSH) terms and free-text keywords, including *“echocardiography,” “univentricular heart,”* and *“Fontan.”*

To further refine the search, additional terms were incorporated, including “hypoplastic left heart syndrome (HLHS),” “Glenn,” “total cavo-pulmonary connection -TCPC- ”, “3D echocardiography,” “4D echocardiography,” “speckle-tracking echocardiography (STE),” “AV valves”, “diastolic function”, “ventricular function”, “thrombus,” and “clot.”

Potentially relevant studies were also identified through manual screening of the reference lists of all eligible articles and review papers, as well as through citation tracking using the Science Citation Index Expanded (Web of Science).

All titles and abstracts retrieved through the search strategy were independently screened for eligibility. Full-text articles were subsequently reviewed when deemed potentially relevant.

Studies were excluded if they: (a) used imaging modalities other than echocardiography; (b) were not published in English; or (c) were published during the late 1980s and 1990s, a period characterized by substantially different imaging technologies and surgical outcomes compared with contemporary Fontan practice.

This review was conducted in accordance with the current guidelines [57].

All selected articles were independently evaluated by three specialists in pediatric echocardiography (M.C., G.D.S, and C.M.). Final inclusion was based on consensus agreement among all reviewers.

This document has been endorsed by AEPC (Association for European Paediatric and Congenital Cardiology) Imaging Working Group (IMWG) committee, a specialist working group, which focuses on cardiac imaging.

The use of anonymized videos and images was approved by the local ethics committee, FTGM CE (approval code: Study “Bet” No. 390). Parents or legal guardians were duly informed, and they provided written informed consent.

## 3. Search Results

A total of 120 publications were initially identified through the search strategy. After title, abstract, and full-text screening, 50 studies met the predefined inclusion criteria and were included in the final analysis.

A total of 70 articles were excluded, including: 15 studies using imaging modalities other than echocardiography, two studies published in languages other than English, and 53 studies published in the late 1980s or 1990s.

## 4. General Aspects

Key elements of the evaluation of univentricular heart across different stages of Fontan palliation include assessments of (Table 1):

(1) Atrial septal and pulmonary venous obstruction;
(2) Ventricular function;
(3) AV valve function;
(4) Aorta and neo-aorta function, subaortic region, and aortic arch;
(5) Pulmonary arteries;
(6) Shunt, Glenn and TCPC conduit (flow velocity, obstruction, thromboembolism);
(7) Assessment of thrombi and clots.

(1) **Pulmonary venous obstruction**

In the univentricular heart (UH), pulmonary venous obstruction may occur either at the level of pulmonary vein to atrial connections or at the level of interatrial septum [1,2,3,4,5]. Pulmonary venous return should be systematically assessed, and obstruction needs to be ruled out, especially in the setting of heterotaxic syndrome where it is more common [1,2,3,4,5].

Obstruction at the level of the interatrial septum is common before the first stage of univentricular palliation and in the interstage period, but it may also occur later [1,2,3,4,5].

The presence of a restrictive patent foramen ovale (PFO) [58,59,60,61,62,63,64] is a well-known risk factor for early and late death after the Norwood procedure [1,2,3,4,5]. Thus, the patency and adequacy in interatrial communication should always be assessed [1,2,3,4,5]. The characteristics of blood flow, blood flow velocity (and derived estimated pressure gradient), septal bulging, and the presence of dilated pulmonary veins are all features which can define a restrictive patent foramen ovale [57,58,59,60,61,62,63]. Various definitions of a restrictive PFO have been described by different authors, including the maximal PFO diameter or the mean Doppler-derived pressure gradient [57,58,59,60,61,62,63].

**Practical advice:**

The presence of pulmonary venous abnormalities should always be systematically assessed, particularly before and after the first stage of UH palliation. Flow turbulence across the foramen ovale should be interpreted with caution and in the context of the overall clinical picture, including oxygen saturation and gas exchange, signs of pulmonary congestion in left-sided obstruction, and hepatomegaly in right-sided disease, in order to avoid misleading qualitative interpretations of echocardiographic findings.

**Figure 1:**
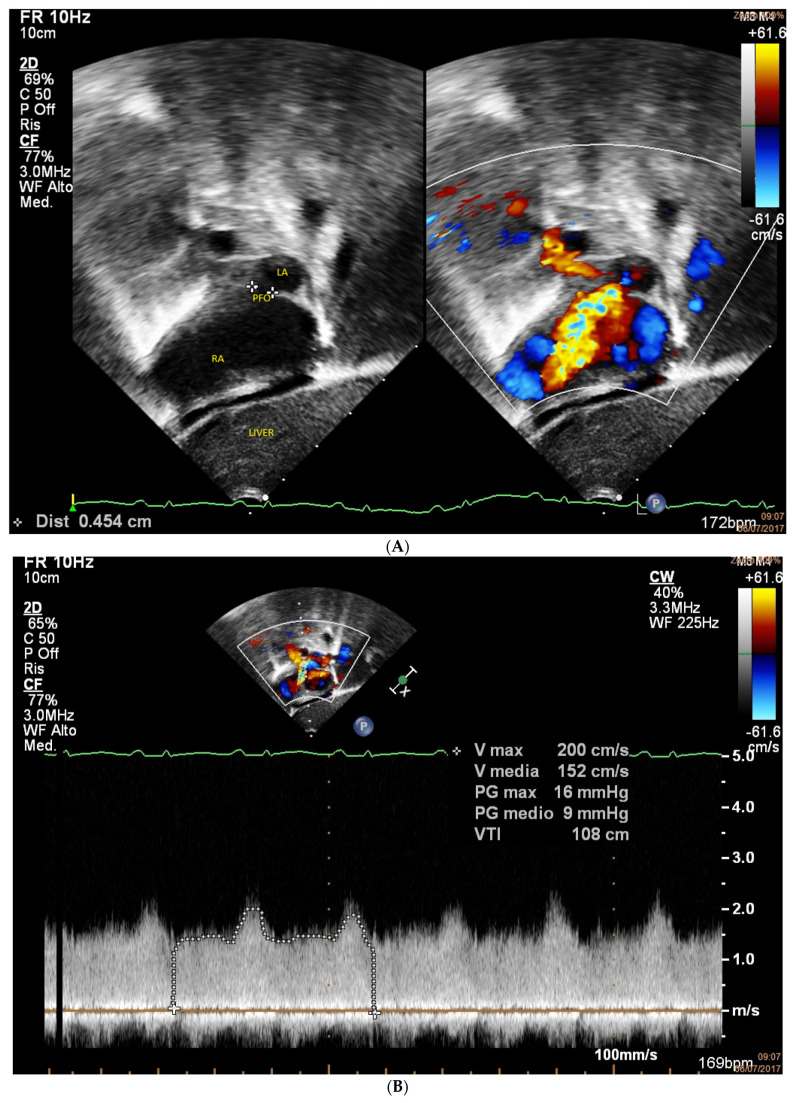
(**A**): subcostal color view of a restrictive (4.5 mm) foramen ovale in a neonate (2 days old, 3 kg of weight) with HLHS; (**B**): gradient across the patent foramen ovale (PFO) (mean gradient 9 mmHg). LA = left atrium, RA = right atrium, PFO = patent foramen ovale.

**Figure 1, Movie S1**

(2) **Evaluation of Systemic Ventricular Function in Univentricular Heart Physiology during Fontan Palliation**

(a) **Systolic Function**

The assessment of systemic ventricular function is a critical component in the evaluation of univentricular heart patients throughout all phases of Fontan palliation [1,2,3,4,5,17,18,19,20,21,22,23,24,25,26,27,28,29,30,31,32,33,34,35,36,37,38,39,40]. The diverse and complex ventricular morphologies in these patients pose challenges for standardizing the evaluation of ventricular function [1,2,3,4,5,17,18,19,20,21,22,23,24,25,26,27,28,29,30,31,32,33,34,35,36,37,38,39,40]. Despite the inherent limitations [27], subjective qualitative assessment, typically categorized as mild, moderate, or severe impairment, remains a commonly used approach [1].

For the systemic right ventricle (RV), a multiparametric echo approach [17,18,19,20,21,22,23,24,25,26,27,28,29,30,31,32,33,34,35,36,37,38,39,40] has been shown to correlate well with cardiac magnetic resonance (CMR) data [20]. These echo parameters include global longitudinal strain (GLS) of the free wall, tissue Doppler velocity of the basal lateral wall (S’), tricuspid annular plane systolic excursion (TAPSE), volumetric function assessed by 3D echocardiography, fractional area change (FAC), and monoplane Simpson ejection fraction (EF) [20].

### 4.1. 2D STE

Excellent correlation between speckle tracking echocardiography (STE) and MRI measurements for both global longitudinal strain (GLS) and regional strain in children with single ventricle physiology has been reported [21]. A significant reduction in both EF and GLS by STE (*p* < 0.01) has been observed in children and adolescents both before and after Fontan surgery, indicating a progressive decline in myocardial function [22,23,30]. Furthermore, STE studies on rotational mechanics have shown statistically significant impairment in both single left ventricle and other forms of single ventricle anatomy.

A progressive decrease in systemic ventricular torsion by STE has correlated with a reduction in apical rotation, which is more pronounced in single right ventricles. This decrease in torsion by STE has been linked to increased markers of myocardial fibrosis, as assessed by T1 mapping in CMR [22,23,24].

### 4.2. 2D STE and 3D Volume

Recent studies utilizing 3D STE have shown that, in HLHS patients post-Fontan, right ventricular GLS is significantly reduced compared to normal controls [34]. Moreover, other authors have highlighted increased ventricular dyssynchrony in children post-Fontan, both by 2D STE [30] and 3D STE [26], with this dyssynchrony being strongly associated with adverse clinical outcomes [26,31].

Additionally, in both children and adults with single ventricle physiology, the ejection fraction and volumes measured by 3D echocardiography have been shown to correlate significantly with cardiopulmonary exercise test variables, such as VO2max (*p* < 0.01) and VE/VCO2 slope (*p* = 0.05) [22,26].

### 4.3. Prognostic Significance of STE GLS

The prognostic significance of GLS measured by STE has been demonstrated in various contexts, including in patients with single left ventricle physiology prior to Fontan [30] and in HLHS patients following the first stage of Fontan palliation [26].

### 4.4. Limitations and Gaps of Evidence of 3D and STE

Reference values for GSL values and 3D volumes from healthy populations may be inappropriate for patients with UH anatomy and physiology, as disease-specific normative data are currently lacking. In addition, these techniques are not always available and may not always be feasible, particularly in young uncooperative children or in the presence of poor acoustic windows.

**Figure 2:**
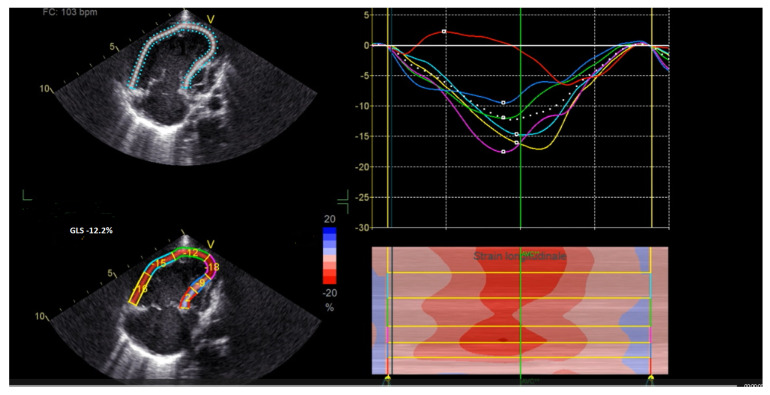
Global longitudinal strain of −12.2%, fractional area change (FAC) 35%, tricuspid annular plane systolic excursion (TAPSE) 8 mm, and tricuspid valve (TV) s’ 8 cm/s.

**Figure 2, Movie S2**

**Practical Advice**

Evaluation of systemic ventricular function is essential throughout all stages of Fontan palliation. A multiparametric approach, integrating conventional 2D echocardiographic measures with advanced techniques, such as STE and 3D parameters when available, is recommended in place of subjective qualitative assessment.

(b) **Assessment of Ventricular Diastolic Function**

Diastolic dysfunction is considered one of the most significant ventricular complications following Fontan palliation [1,2,3,4,5], with an incidence up to 72% of Fontan patients in a large cohort study [30]. A classification system for diastolic dysfunction (normal, impaired relaxation, pseudo normal, and restrictive) in Fontan patients has been proposed [30].

However, this grading system, derived from adult data, lacks validation against invasive pressure measurements in both pediatric and adult Fontan patients and of correlation with clinical outcomes [30]. Invasive studies attempting to link diastolic echocardiographic parameters with direct pressure measurements have also failed to yield useful clinical associations [2,5,27]. It is important to note that not only have adult parameters for diastolic function not been validated in the pediatric age but also most echocardiographic markers of diastolic function, such as AV valve inflow, pulmonary venous flow, and tissue Doppler imaging which are influenced by variables like heart rate, loading conditions, AV valve size and function, and univentricular heart systolic performance [2,27].

In aging Fontan patients [40], progressive ventricular stiffness and elevated end-diastolic pressures are frequently observed. However, these changes are challenging to detect through noninvasive methods. In the general adult population with biventricular circulation, the E/E′ ratio is commonly used as an indicator of elevated filling pressures [2,27]. However, its applicability in patients with univentricular physiology is limited due to the influence of atrioventricular (AV) valve size on E velocity and the frequent preservation of E′ velocity. While diastolic dysfunction plays a critical role in Fontan failure, current echocardiographic techniques lack reliability in identifying at-risk patients based on existing diagnostic criteria [2,40]. Even invasive assessment in the catheterization lab remains challenging, as filling pressures are highly dependent on intravascular volume status [2,40]. Detecting single ventricle physiology patients with reduced ventricular compliance remains a significant challenge in congenital cardiology. Future advancements, such as MRI-based T1 mapping for diffuse fibrosis assessment and myocardial stiffness evaluation via ultrafast ultrasound, may enhance the understanding of tissue characteristics and improve diagnostic precision [2,40].

**Atrial strain**

Several studies have investigated the potentialities of STE in the evaluation of atrial function in Fontan circulation, particularly its relationship with ventricular filling pressures [41], cardiac output [42], and functional capacity [44]. A progressive decrease in atrial function through different stages of Fontan palliation has been identified [44]. Data on atrial STE data and their correlation with hemodynamic parameters are controversial. Despite atrial strain values in Fontan patients poorly correlating with invasive ventricular filling pressures (R^2^ = 0.12) [41], atrial reservoir strain value [42] was associated with decreased cardiac index and adverse post-Fontan clinical outcomes (R^2^ = 0.28). Furthermore, a positive correlation (R^2^ = 0.34) between atrial strain and exercise performance in Fontan patients [43] has been demonstrated. These studies [41,42,43] collectively underscore the importance of atrial function in Fontan physiology and its potential role in predicting adverse outcomes, functional status, and hemodynamic efficiency. However, further research is needed to establish standardized clinical applications of atrial strain measurements in this population.

*Limitations and gaps of evidence of atrial STE strain:* Reference values derived from healthy populations may be inappropriate for patients with UH physiology, as disease-specific normative data are currently lacking. In addition, STE is not always available and may not always be feasible, particularly in young uncooperative children or in the presence of poor acoustic windows.

**Practical advice:** Echocardiographic assessment of diastolic function should always be attempted, despite limitations related to the lack of validated parameters and load dependency. In the absence of standardized reference values for the UH population, longitudinal assessments of trends are preferable to the reliance on single cut-off measurements, and findings should always be interpreted in conjunction with clinical and hemodynamic data. STE atrial strain analysis may provide additional useful information, although larger studies are needed for validation.

(3) **Assessment of Atrioventricular Valve Regurgitation**

Atrioventricular (AV) valve regurgitation is a common complication of significant prognostic implications in Fontan patients, particularly tricuspid regurgitation (TR) in HLHS [1,2,3,4,5,7,8,9,10,11]. Severe TR in HLHS can lead to right ventricular dysfunction, poor hemodynamics, and reduced exercise capacity, all of which contribute to a worse clinical outcome [9]. The severity of TR is often correlated with impaired ventricular function, particularly in the systemic right ventricle (RV) in patients with HLHS and Fontan circulation [22]. Echocardiographic assessment is crucial in diagnosing and monitoring the progression of AV valve regurgitation and determining its impact on clinical outcomes [1,2,3,4,5,7,8,9,10,11]. The complexity of AV valve morphology in univentricular heart necessitates a detailed and precise evaluation using advanced imaging techniques [1,2,3,4,5,7,8,9,10,11].

### 4.5. Echocardiographic Techniques for Assessing AV Valve Regurgitation

*Two-Dimensional Echocardiography (2D Echo):* Traditional 2D echocardiography has been the cornerstone for assessing AV valve function. In HLHS, it allows visualization of the tricuspid valve (TV) structure and the identification of regurgitant jets. However, 2D echo has limitations in assessing the severity of regurgitation due to the dynamic nature of the valve and regurgitant flow [1,2,3,4,5,7,8].

*Doppler Imaging:* This plays a key role in assessing the regurgitant jet of the tricuspid valve. Color Doppler allows for the identification of the regurgitant jet direction, while pulsed-wave Doppler measures the velocity and timing of the regurgitant flow [1,2,3,4,5,7,8]. Unfortunately, at present, quantitative and semiquantitative parameters to evaluate AV regurgitation in children are limited, and adult criteria have been often adopted without validation [1,2,3,4,5,7,8,27]. An example of a qualitative TR grading system is as follows: trivial (no regurgitation or a single, narrow jet), mild (multiple narrow jets), moderate (a broad jet extending to the mid-portion of the right atrium), and severe (a broad jet reaching the posterior wall of the right atrium).

*3D Echocardiography* offers superior visualization of AV valve morphology and provides a more accurate assessment of regurgitation [8,10,11]. In HLHS patients, 3D echo allows for a detailed evaluation of the tricuspid valve annulus, leaflet motion, and the degree of tethering, which is essential in understanding the functional impact of regurgitation [11]. Studies have demonstrated that 3D echocardiography can identify the location and severity of TR, which is critical for preoperative planning and monitoring post-surgical outcomes [8].

In HLHS, moderate or greater TR is often associated with structural abnormalities of the tricuspid valve, including increased leaflet billow volume, an enlarged tricuspid annulus, and altered papillary muscle orientation [7]. These abnormalities can lead to significant leaflet tethering, a key contributor to persistent TR after surgery [8]. 3D echo imaging has been instrumental in identifying these structural changes, providing a more comprehensive understanding of the pathophysiology of TR.

Annular dilation is a hallmark feature of AV valve regurgitation in HLHS, particularly in the context of Fontan physiology. 3D echo has been shown to better assess the extent of annular dilation compared to 2D echo, allowing for more accurate surgical planning [10,11]. Furthermore, leaflet prolapse and tethering are frequently observed in patients with severe TR [10,11]. Anterior leaflet prolapse represents the most common mechanism of TR, followed by septal leaflet prolapse or tethering [11]. Preoperative imaging that identifies these abnormalities can guide the surgical decision-making process, often requiring individualized planning [7,8,9,10,11]. Traditional surgical techniques, such as posterior annuloplasty and commissuroplasty, are commonly used to address annular dilation and posterior leaflet abnormalities [7,8,9,10,11]. However, these techniques may not fully correct septal leaflet tethering, a key factor contributing to persistent regurgitation [8]. As such, image-guided surgical planning based on 3D echo and Doppler studies is becoming increasingly important to optimize outcomes in this high-risk population [7].

### 4.6. Limitations and Gaps of Evidence of 3D Echocardiography

Reference values for atrioventricular (AV) valve dimensions derived from healthy populations may be inappropriate for patients with UH physiology, as disease-specific normative data are currently lacking. In addition, 3D echocardiography is not always available and may not always be feasible, particularly in young uncooperative children or in the presence of poor acoustic windows.

**Practical advice:** Assessment of the AV valve represents a fundamental component of the echocardiographic evaluation in UH patients throughout all stages of Fontan palliation. In the absence of standardized quantitative parameters for regurgitation assessment, serial evaluation should rely on qualitative and semi-quantitative descriptors, including the number and intra-atrial extent of regurgitant jets, as well as annular dilatation, leaflet tethering, and prolapse, together with ventricular areas and volumes. Three-dimensional echocardiographic assessment may be particularly useful in the presence of at least moderate regurgitation, when a more detailed understanding of valve anatomy and a more accurate quantification of regurgitation severity are required.

**Movie S3**

(4) **Aorta, neo-aorta, and aortic arch**

In the univentricular heart, the dominant ventricle’s outflow tract connects to the aorta. If an obstruction is present, it leads to chronic pressure overload and secondary concentric hypertrophy, negatively affecting long-term ventricular function [1,2,3,4,5,12]. This can further limit cardiac output and worsen diastolic dysfunction [1,2,3,4,5,12].

*Outflow tract:* Outflow tract obstruction is typically subvalvular, as seen in conditions like double-inlet left ventricle with transposition of the great arteries, where a restrictive ventricular septal defect (VSD) can cause subaortic obstruction [13,14,15,16]. When a Damus–Kaye–Stansel (DKS) is present, echocardiographic imaging is crucial for detecting any obstruction, particularly in patients who have not undergone a DKS procedure [1,2,3,4,5,13,14,15,16]. Various acoustic windows (subxiphoid, apical, parasternal long-axis) are used, with Doppler techniques employed to measure pressure gradients and identify obstruction mechanisms, such as restrictive VSDs, fibrous rings, or valvular stenosis [1,2,3,4,5,13,14,15,16].

**Movie S4**

**Movie S5: DKS**

*Native aorta:* Assessment of native aorta is also important, since it represents the source of flow for coronary arteries [1,2,3,4,5,48,49,50,51]. The patency and adequacy of native aorta–neo-aorta communication are of paramount importance. The DKS connection must also be evaluated, as obstruction at this site can impair coronary perfusion and lead to ischemia and ventricular dysfunction [1,2,3,4,5,48,49,50,51].

*Aortic root:* Assessment of the aortic root includes measurements of its size and morphology. After extensive aortic reconstruction, progressive dilation of the aortic root and ascending aorta can occur, potentially leading to aneurysm formation and requiring reintervention [1,2,3,4,5,12].

*Aortic arch*: In patients with a history of aortic arch reconstruction or coarctation surgery, it is essential to assess residual arch obstruction, which can cause hypertension and increased afterload on the single ventricle [1,2,3,4,5]. Even mild gradients across the arch can contribute to ventricular dysfunction and long-term diastolic impairment [1,2,3,4,5]. The suprasternal window is optimal for imaging the aortic arch, with Doppler techniques used to measure gradients [1,2,3,4,5,27]. In cases where arch imaging is challenging, pulsed-wave Doppler of the abdominal aorta from the subxiphoid window can help exclude significant residual obstruction [1,2,3,4,5].

**Practical advice:** Evaluation of the neo-aortic valve, the native aorta, and the subaortic region is essential throughout all phases of Fontan palliation. In addition, aortic root dilatation should be carefully assessed during long-term follow-up.

(5) **Pulmonary Arteries**

Echocardiography plays an essential role for the evaluation of pulmonary artery size assessment and its quantification (through z-score), to identify the presence of narrowing and stenosis, and to evaluate the flow. In patients after different stages of Fontan palliation, it is important to evaluate the redistribution of flow within the pulmonary arteries from different sources including BTM shunt, Glenn, and TCPC conduit [1,2,3,4,5,6]. The presence of competitive flow from a native pulmonary artery left partially patent needs which should also be evaluated [1,2,3,4,5,6].

**Practical advice:** Evaluation of pulmonary artery size and flow is essential throughout the different stages of UH palliation. The presence of stenosis is of paramount importance, particularly during the first two stages of palliation. In the Glenn stage, competitive flow between antegrade pulmonary blood flow and Glenn flow should be carefully assessed.

(6) **Shunt, RV/Single Ventricle–PA Conduct, Glenn, and TCPC Conduct**

## 5. Blalock–Taussig-Modified (BTM) Shunts

A BTM shunt is a crucial palliative surgical procedure used in infants with single ventricle physiology to provide pulmonary blood flow until definitive staged palliation, such as a bidirectional Glenn or Fontan procedure, can be performed [1,2,3,4,5,65,66,67,68]. This shunt consists of a Gore-Tex^®^ polytetrafluoroethylene (PTFE) conduit connecting the systemic circulation (typically the subclavian or innominate artery) to the pulmonary artery (PA), ensuring pulmonary perfusion in patients with inadequate or absent native pulmonary blood flow [1,2,3,4,5,65,66,67,68].

### 5.1. Hemodynamics and Flow Characteristics

The flow pattern in a BTM shunt is continuous and non-pulsatile, driven by systemic arterial pressure rather than the right ventricle [1,2,3,4,5,65,66,67,68]. Unlike normal pulmonary circulation, which is pulsatile and dependent on the right ventricular systole, the BTM shunt delivers a steady but pressure-dependent flow into the pulmonary arteries [1,2,3,4,5,65,66,67,68]. This leads to a diastolic flow reversal in the aortic arch, as systemic diastolic pressure is partially reduced due to the run-off into the pulmonary circulation [1,2,3,4,5,65,66,67,68]. This can contribute to coronary and systemic hypoperfusion, particularly in neonates with parallel systemic and pulmonary circulations [1,2,3,4,5,65,66,67,68]. In univentricular heart with BTM, the pulmonary perfusion is shunt-dependent, which may lead (especially in first pre-operative weeks) to an excessive pulmonary flow, pulmonary over-circulation, volume overload, and heart failure, while inadequate flow can result in hypoxemia and cyanosis [1,2,3,4,5,65,66,67,68]. Several factors influence the efficiency and balance of pulmonary blood flow through the BTM shunt. These include shunt size, systemic pulmonary vascular resistance, and patient growth. A larger shunt increases pulmonary flow but raises the risk of steel syndrome, reducing systemic perfusion including coronary and cerebral circulation [1,2,3,4,5,65,66,67,68].

**Supplemental Movie S1**

Conversely, a small shunt may lead to inadequate oxygenation and hypoxia [1,2,3,4,5,65,66,67,68]. Changes in PVR and systemic vascular resistance (SVR) significantly alter shunt flow [1,2,3,4,5,65,66,67,68]. High PVR (e.g., due to lung disease or hypoxia) reduces pulmonary perfusion, while low PVR can lead to excessive pulmonary flow and heart failure [1,2,3,4,5,65,66,67,68]. As the infant grows, a fixed-size shunt may become progressively restrictive, necessitating shunt revision or progression to the next surgical stage [1,2,3,4,5,65,66,67,68].

*Flow velocity* and turbulence of the BTM are typically high, reflecting the pressure difference between systemic (aorta) and pulmonary (pulmonary arteries) circulation. BTM velocities are influenced by shunt size, systemic resistance, and pulmonary vascular resistance (PVR) [1,2,3,4,5,65,66,67,68].

**Movie S6**

**RV/Single Ventricle–PA Conduct**

The Sano modification of the Norwood procedure is a surgical technique used in single ventricle heart physiology, particularly in HLHS [1,2,3,4,5]. This approach utilizes a right ventricle-to-pulmonary artery (RV-PA) conduit, often made of a non-valved Gore-Tex (W. L. Gore & Associates, Newark, DE, USA)^®^ tube, to provide pulmonary blood flow while reducing diastolic run-off and improving coronary perfusion compared to the traditional BT shunt [1,2,3,4,5,65,66,67,68].

### 5.2. Hemodynamics and Flow Characteristics in Sano Conduit, Glenn Anastomosis and in Fontan Conduit

The flow pattern in the Sano conduit differs significantly from other systemic-to-pulmonary shunts due to its direct connection between the right ventricle (RV) and pulmonary arteries (PAs) [1,2,3,4,5,65,66,67,68]. Key characteristics of Sano conduit flow include a pulsatile flow to the pulmonary arteries (unlike the continuous, pressure-driven flow in BT shunts) resembling native right ventricular output, which may promote better pulmonary artery growth [1,2,3,4,5].

In the past, non-reinforced RV-PA conduits used for the Sano modification were susceptible to stenosis (Movie S7). Nowadays, reinforced conduits are routinely employed, which are less likely to become stenotic. The flow in the Sano conduit has systolic dominance with diastolic flow reversal (Movie S8). The diastolic regurgitation is a potential problem in Sano palliation since it may lead to an increased RV volume load, reducing systemic cardiac output and potentially contributing to ventricular dysfunction over time [1,2,3,4,5,65,66,67,68].

Several factors influence the efficiency and long-term outcomes of pulmonary blood flow through the Sano conduit, including conduit size and length. A larger conduit allows for increased pulmonary blood flow but may exacerbate diastolic regurgitation and ventricular volume overload [1,2,3,4,5,65,66,67,68]. A smaller conduit instead may restrict pulmonary blood flow, leading to hypoxia and insufficient pulmonary perfusion [1,2,3,4,5,65,66,67,68]. The RV in the Sano circulation must sustain a chronic systolic (sustain both the systemic and pulmonary circulation) and diastolic overload (diastolic regurgitation through the conduit) which can contribute to ventricular dilation and failure, especially in the long term [1,2,3,4,5,65,66,67,68]. The presence of a stenosis at the level conduit may exacerbate the systolic overload leading to acute systolic dysfunction [1,2,3,4,5,65,66,67,68].

The Sano conduit may be appreciated by echocardiography in different projections (subcostal, modified 4 chambers, short axis). At times, echocardiographic assessment of the Sano conduit may be difficult by echocardiography since it lies in the retrosternal area, an area which is difficult to examine, especially in the presence of medication (after surgery) or thoracic deformities [1,2,3,4,5,64,65,66,67]—often just the proximal part of the conduit is visible [55,56]. The use of a linear probe may allow a better assessment of the retrosternal area and visualization of the conduit through all its length [55,56].

**Glenn**

The Glenn anastomosis, a key component of staged palliation for single ventricle physiology, involves the direct connection of the superior vena cava (SVC) to the pulmonary arteries, bypassing the right atrium [1,2,3,4,5,65,66,67,68]. This procedure establishes a passive pulmonary circulation that relies on systemic venous pressure to drive pulmonary blood flow [1,2,3,4,5,65,66,67,68]. The hemodynamic patterns in the Glenn circulation are influenced by multiple factors, including central venous pressure, pulmonary vascular resistance, and the presence of collateral flow [1,2,3,4,5,65,66,67,68].

The Glenn shunt creates a non-pulsatile, low-pressure pulmonary blood flow, in contrast to the pulsatile flow present in a normal pulmonary circulation [1,2,3,4,5,65,66,67,68]. The absence of a right ventricular pump means that flow is predominantly dependent on venous return dynamics and respiratory mechanics, with inspiration lowering intrathoracic pressure and promoting forward flow into the pulmonary arteries [1,2,3,4,5,65,66,67,68]. Expiration and elevated intrathoracic pressure may transiently reduce pulmonary blood flow due to increased venous resistance [1,2,3,4,5,65,66,67,68].

For imaging Glenn connections [1,2,3,4,5], suprasternal views are preferred, and at times apical and short axis views may be employed. Color Doppler with low Nyquist settings should be used to assess surgical anastomoses [1,2,3,4,5]. The flow in the Glenn anastomosis is typically phasic with fluctuations corresponding to both the cardiac cycle and respiratory cycle, where a respiratory-dependent component becomes particularly significant [1,2,3,4,5].

The distribution of blood flow between the right and left pulmonary arteries depends on several anatomical and physiological factors, including the angle of the anastomosis, as well as the size and compliance of the pulmonary arteries [1,2,3,4,5,65,66,67,68]. Although the Glenn anastomosis provides a stable pulmonary circulation, certain flow disturbances can arise including the development of venous collateral formation (leading to inefficient flow patterns and systemic desaturation), flow imbalance between pulmonary arteries (uneven flow distribution can result from pulmonary artery hypoplasia, stenosis, or anastomotic angulation), and increased pulmonary vascular resistance (PVR) that can impair passive blood flow, increasing systemic venous pressures and contributing to venous congestion and Fontan failure in later stages [1,2,3,4,5,64,65,66,67].

**Figure 3:**
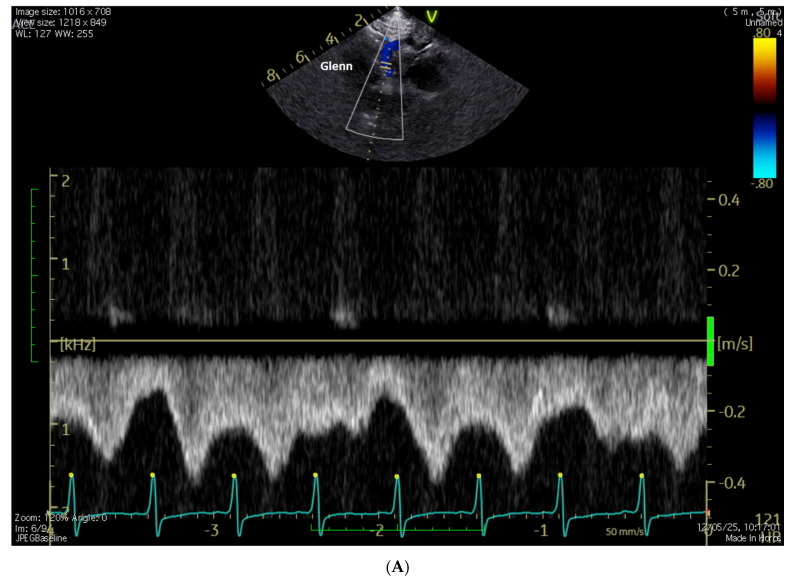

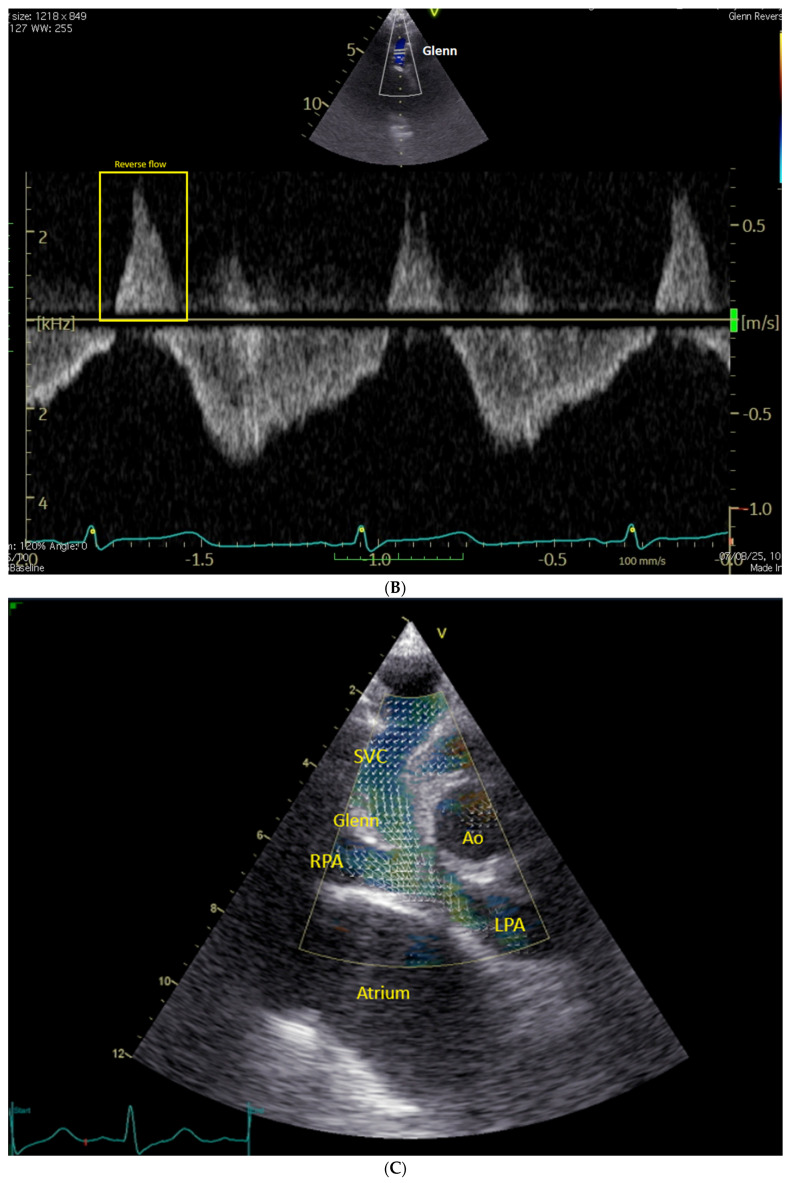
(**A**): normal, phasic, and low-velocity Doppler flow in Glenn; (**B**): reverse flow in Glenn; (**C**): blood flow in Glenn and pulmonary arteries are visualized by BST vector flow reconstruction. Ao = aorta, LPA = left pulmonary artery, RPA = right pulmonary artery, SVC = superior vena cava.

**Figure 3 and Movie S9**

TCPC in Fontan circulation directs systemic venous blood from the SVC and inferior vena cava (IVC) directly to the pulmonary arteries, bypassing the ventricle. This configuration optimizes hemodynamics by reducing energy loss and improving pulmonary blood flow distribution, which is critical for long-term Fontan physiology [65,66,67,68]. TCPC in Fontan circulation can be achieved through different surgical techniques. The lateral tunnel approach utilizes an intracardiac baffle to direct IVC blood to the pulmonary arteries, while the extracardiac conduit (or intra-atrial–extracardiac tunnel) employs an external graft to achieve the same goal [65,66,67,68].

Coronal and subxiphoid sagittal views provide optimal visualization of the IVC and its connection to Fontan baffle. Color flow imaging and pulsed-wave Doppler are used to assess obstructions [1,2,3,4,5,68]. Typically, the IVC and hepatic veins appear dilated with low-velocity flow (<20–30 cm/s) and spontaneous contrast. A normal flow pattern consists of continuous systolic and diastolic flow with respiratory variation, indicating unobstructed Fontan connections [1,2,3,4,5,69] (Table 2).

The absence of respiratory variation or the presence of retrograde flow suggests obstruction, AV regurgitation, or competitive collateral circulation [1,2,3,4,5,69]. Flow reversal during diastole may indicate failing Fontan physiology or atrial arrhythmia. The intra-atrial tunnel or extracardiac conduit can be assessed using subxiphoid long- and short-axis sweeps [1,2,3,4,5]. Additional views, including apical and parasternal, aid in evaluating tunnel dilation, thrombi, fenestrations, or leaks. If a fenestration is present, pulsed Doppler can determine the transpulmonary gradient, and for closed fenestrations, device positioning and residual shunting should be assessed [1,2,3,4,5,69].

**Figure 4:**
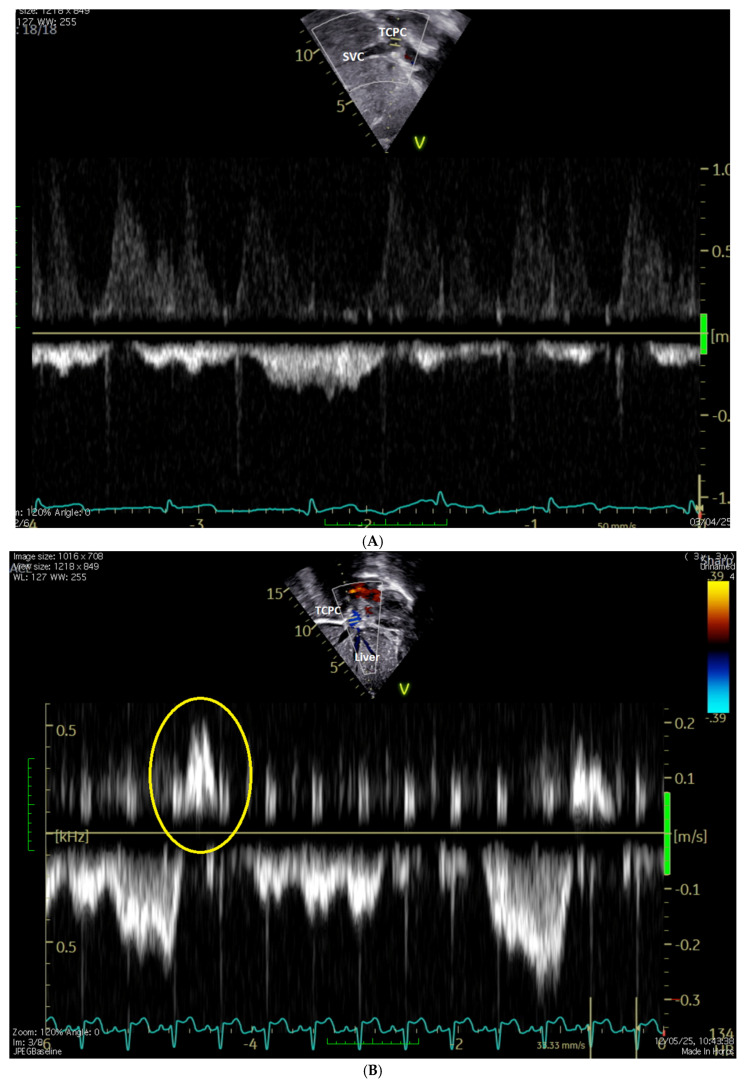

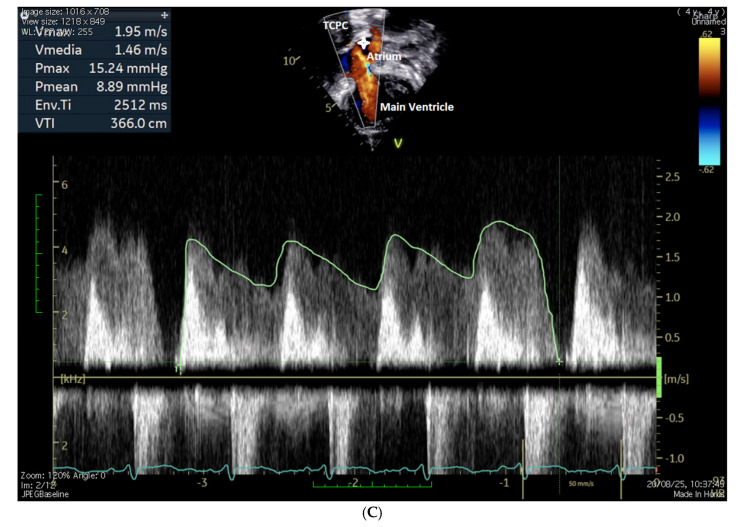
(**A**): Doppler of normal, phasic, and low-velocity (max velocity 0.4 m/s, mean velocity 0.2 m/s) TCPC conduct with respiratory variation; (**B**): Doppler of TCPC conduct with mild reversal; and (**C**): fenestrated TCPC with the gradient across the fenestration. TCPC = total cavo-pulmonary connection conduct; fenestration is arrowed.

**Figure 4 and Movie S10**

**Practical advice:**

Assessment of the BT shunt/Sano conduit, Glenn, and TCPC conduit is essential across all stages of Fontan palliation. Echocardiographic evaluation should focus on both anatomical patency and flow characteristics, including flow direction, the presence of turbulence or stasis, and velocities/gradients.

(7) **Risk of Thrombus and Clot Formation in Fontan Physiology: The Role of Echocardiographic Assessment**

The Fontan procedure is a complex surgical intervention for patients with univentricular hearts. Although this approach improves survival and quality of life, it also introduces new risks, including thrombus and clot formation [46,47]. **It is crucial to distinguish between these two types of abnormalities.**

*Thrombus* is a pathological blood clot that forms within blood vessels or cardiac chambers and may develop at any stage along the Fontan pathway as a consequence of blood stasis. *Clot*, in contrast, generally refers to extravascular coagulated blood, typically forming postoperatively as a result of bleeding outside the heart, such as in the retrosternal space, and usually (although not invariably) surrounding the cardiac chambers.

Both thrombi and clots may be diagnosed and followed with transthoracic echocardiography [46,47].

(8) **Possible Site of Thrombus Formation in Fontan Circulation**

In patients with Fontan physiology, thrombus formation is most observed in several anatomical locations due to hemodynamic alterations that contribute to stasis and hypercoagulability [46,47].

***Native aorta:*** Native aortic thrombosis in HLHS is a rare, serious, and underrecognized complication that may occur at different stages of Fontan palliation [46,47]. The abnormal flow in this vessel, especially post-Fontan, creates an environment conducive to thrombosis, with regions of low or turbulent blood flow being particularly vulnerable [8]. Native aortic thrombosis can lead to left ventricular (LV) failure, arrhythmias, myocardial ischemia, and death [48,49,50,51]. Presentation widely varies from incidental findings [48,49,50,51] in totally asymptomatic children to severe symptoms due to myocardial ischemia up to sudden death [48,49,50,51]. The most common presentation was chest pain (accompanied with ECG abnormalities and troponin increase) or arrhythmic events. Diagnosis is generally feasible with transthoracic echocardiography [48,49,50,51,69], but experience and a careful and systematic assessment of the native aorta are required. The increased awareness of this rare complication has led to an increasing diagnostic rate in the past few years with multiple cases described [48,49,50,51,69].

**Figure 5:**
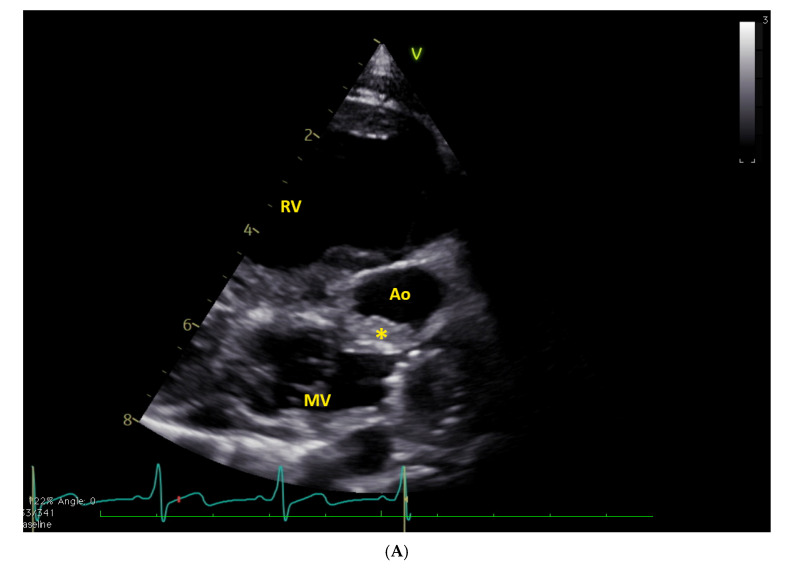

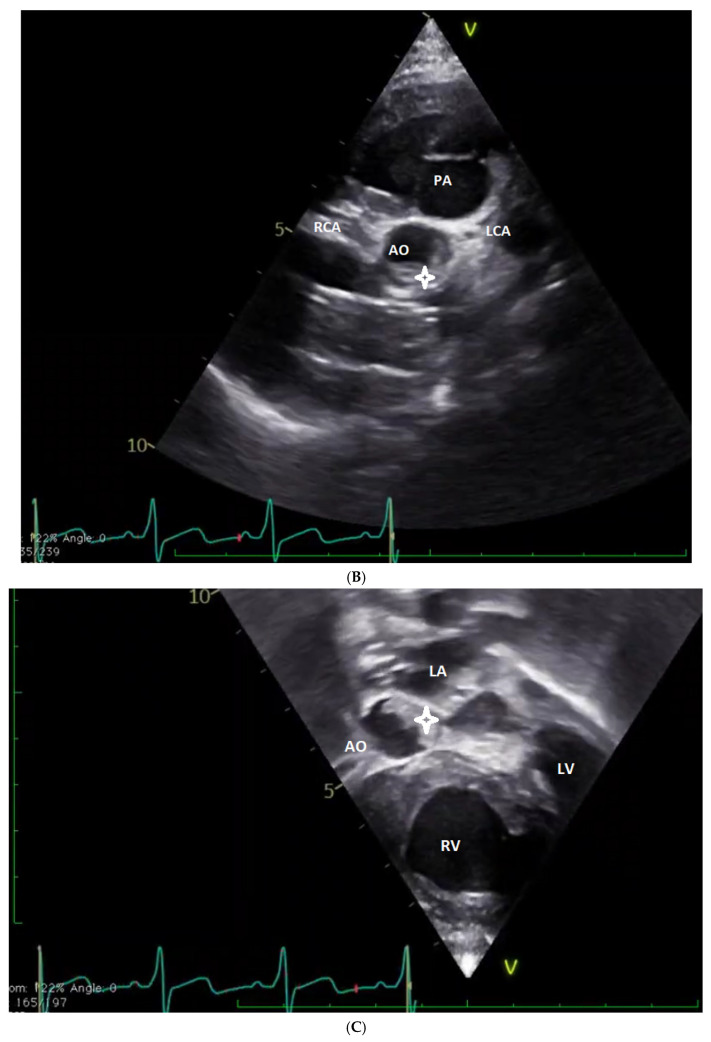
A 3-year-old with HLHS after Glenn operation with native aorta thrombosis visualized in long-axis view (asterisk) (**A**), modified 5-chamber view (star) (**B**), and short-axis view (star) (**C**). The clot is indicated with arrow. Ao = aorta, LA = left atrium, LV = left ventricle, RV = right ventricle, PA = pulmonary artery.

**Movie S11, Figure 5**

***Hypoplastic Left Ventricle:*** In patients with HLHS who undergo Fontan palliation, the hypoplastic LV is functionally inefficient, resulting in a stagnation of blood flow and a predisposition to thrombus formation [52,53,54]. Despite being rare, thrombus within the HLHS have been described and can be diagnosed by echocardiography [52,53,54].

**Figure 6:**
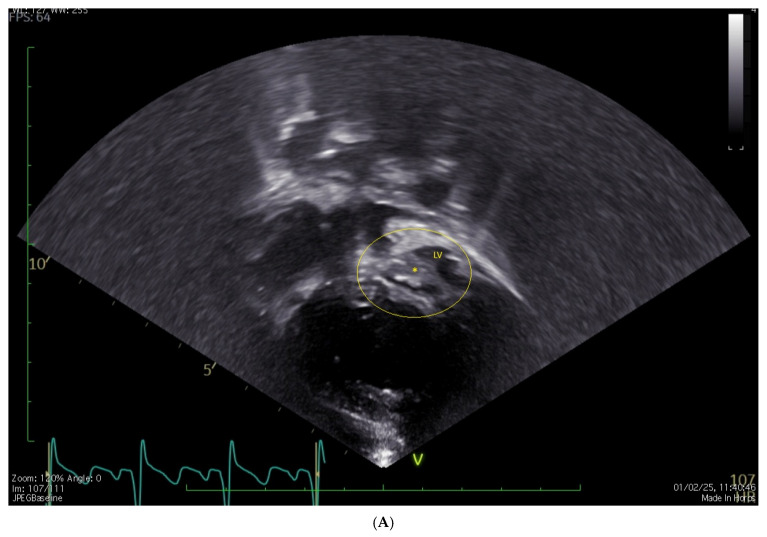

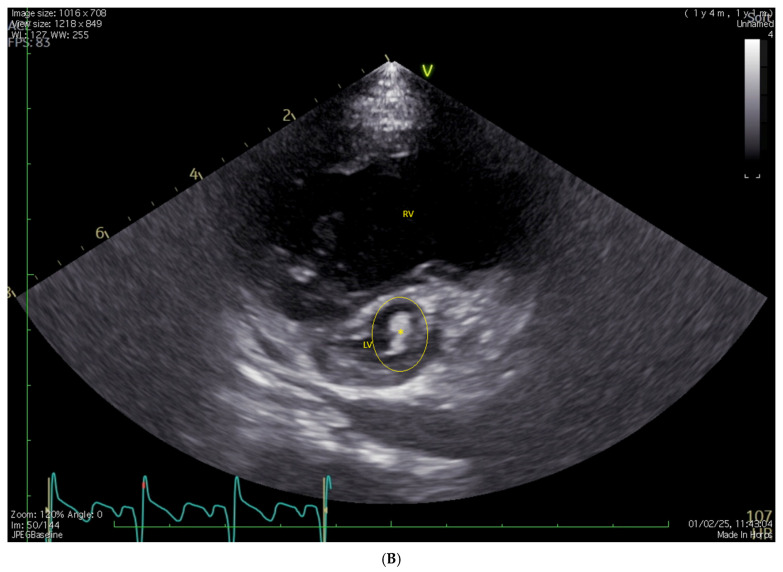
(**A**): a 4-chamber view, and (**B**): a short-axis view of a thrombus (asterisk) in LV in a HLHS. LV = left ventricle, RV = right ventricle.

**Movie S12 and Figure 6**

***Inferior Vena Cava (IVC):*** The IVC is another common location for thrombus formation, particularly in the context of Fontan physiology. The Fontan procedure leads to venous congestion and reduced flow in the IVC, contributing to venous stasis, which is a key risk factor for thrombus formation. Thrombi in the IVC may extend into the Glenn shunt or Fontan circuit, leading to a higher risk of systemic embolism [1,2,3,4,5,46,47]. Transthoracic echocardiography has been used to visualize thrombus formation in the IVC, with Doppler studies providing valuable information about flow dynamics and the presence of thrombi [1,2,3,4,5,46,47].

**Movie S13**

***BTM shunt, Glenn, and TCPC Conduit:*** Thrombi can also form in the BTM shunt, Glenn, or TCPC conduit, particularly when there is stasis of blood flow or structural abnormalities in these vessels [67,68,69]. These regions are prone to thrombus formation due to the passive flow conditions post-surgery, and echocardiography is pivotal in detecting thrombi in these locations early, preventing further complications [1,5,46,47].

**Figure 7:**
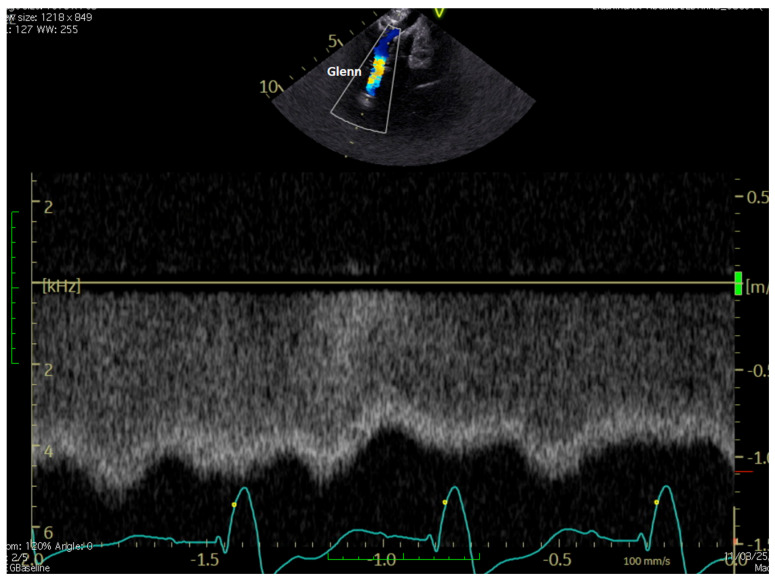
Images showing the non-phasic, continuous but significantly accelerated (v max 1 m/s), flow across the mildly stenotic Glenn conduit.

**Movie S14, Figure 7**

(9) **Post-operative Clot Formation after Glenn and TCPC**

A clot refers to extravascular coagulated blood that typically forms in the early postoperative period as a consequence of bleeding outside the heart, most commonly in the retrosternal space and usually, although not invariably, surrounding the cardiac chambers [55,56]. Clots are often detected incidentally during routine echocardiographic examinations and are particularly common after Glenn and Fontan surgeries [55,56].

**Figure 8:**
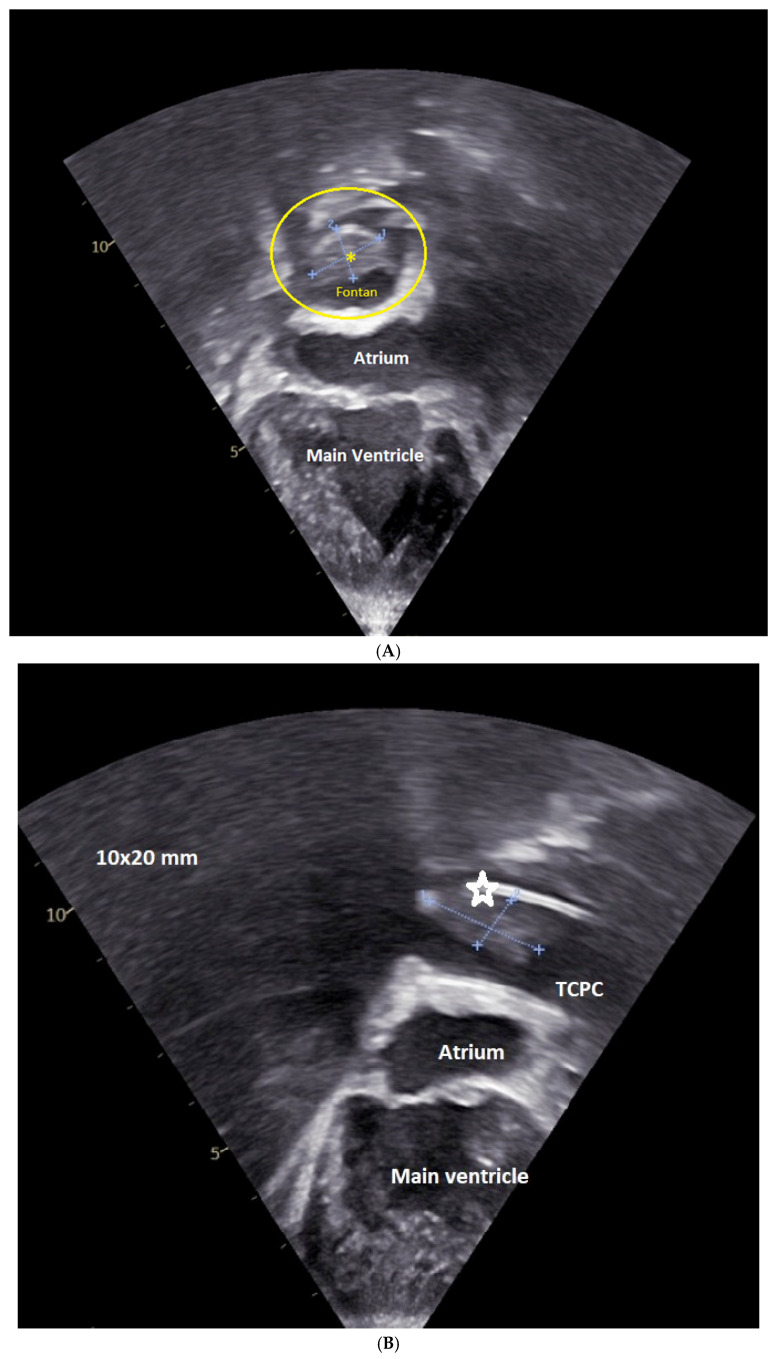
Thrombus in TCPC conduct visualized in a 4-chamber view (asterisk) (**A**); and in a modified 4-chambers view (star) (**B**) of its extension.

Echocardiography plays a critical role in detection, monitoring, and follow-up of clot after surgery. Clots may be assessed either by phase array probes using conventional subcostal and parasternal projections (Figure 8, Movie S15) or by a linear probe [55,56].

For clot assessments, the retrosternal area must be systematically evaluated by use of a linear probe [55,56]. The probe needs to be placed close to the parasternal line, and the anterior segments scanned up and down. When a clot or hematoma is suspected, the linear probe needs to be placed over the mass and freely tilted in various planes or orientations to obtain its visualization [55,56]. Clot sizes have been arbitrarily defined according to the maximal diameter on an axis perpendicular to the cardiac wall—(1) large clots: >3 cm; (2) moderate-sized clots: >2 to <3 cm; (3) small-to-moderate-sized clots: >1 to <2 cm; and (4) small clots: <1 cm [55,56]. However, this classification represents an oversimplification, since the clot size needs to be related to the patient’s body size [55,56].

### 5.3. Limitations and Gaps of Evidence in the Evaluation of Clots

The use of a linear probe for the evaluation of retrosternal is advised; however, projections have not yet been standardized. Quantification has also not been standardized according to projections and the patient’s body size. Furthermore, the presence of medication after sternotomy may limit the parasternal acoustic window.

**Movie S16**

**Practical advice:** The presence of thrombi should always be actively excluded in UH patients across all stages of Fontan palliation. Common sites of thrombosis include hypoplastic ventricles, the neo-aorta, the inferior vena cava, BT shunts, the Glenn pathway, and the TCPC conduit. Thrombus formation is relatively frequent following Glenn and TCPC procedures and should be systematically ruled out. In this context, the use of a high-frequency linear probe with parasternal views may be helpful for improved visualization.

**Reporting FAC sheets**

Practical examples of how reporting in children at different stages of UH palliation are provided in the Supplemental Tables S1–S5 [27]. These formats may also serve as a guide for a sequential analysis and as a reminder of the essential points to be assessed, helping to avoid omission of important information [27].

**Conclusive remarks**

We present a comprehensive practical guidance for the systematic echocardiographic assessment of the univentricular heart across the various stages of univentricular palliation, with particular emphasis on the role of advanced 3D and 4D imaging techniques and the evaluation of thromboembolic complications. This work serves as a foundational reference for the echocardiographic evaluation of univentricular hearts and paves the way for further research aimed at addressing unresolved challenges, such as the assessment of diastolic function in Fontan patients and the complex flow dynamics within Glenn and TCPC conduits.

## Figures and Tables

**Table 1:** Key elements to be searched at different stages.

	*Pre-* *Norwood*	*Interstage*	*Pre-* *Glenn*	*Pre-* *Fontan*	*Post-* *Fontan*
*Atrial septum and pulmonary venous obstruction*	xxx	xxx	xx	x	x
*Ventricular function*	xxx	xxx	xxx	xxx	xxx
*AV valve function*	xx	xx	xx	xxx	xxx
*Aorta and neo-aorta function, subaortic region*	xx	xx	xx	xxx	xxx
*Aortic arch*	xxx	xxx	xx	x	x
*Shunt/Sano conduit*		xxx			
*Glenn*			xxx	xx	xx
*TCPC conduit*				xxx	xxx
*Thrombi and clots*		xxx	xxx	xx	xxx

x: important; xx very important; xxx essential.

**Table 2:** Flow characteristics across Glenn and Fontan (TCPC) circuit.

Circuit	Site	Normal Peak Velocity (m/s)	Flow Dynamic
Bidirectional Glenn	SVC to PA	<0.5 *	Non-pulsatile (or mildly phasic with respiration), antegrade
Fontan (TCPC)	Extracardiac conduit	0.2–0.5 °	Respiratory variation
	Lateral tunnel	0.2–0.5 °	Similar to conduit
	Hepatic veins	<0.5	Forward flow phasic with respiration; reversal suggests dysfunction

PA = pulmonary artery, SVC = superior vena cava, TCPC = total cavo-pulmonary conduit. * Higher velocities (>0.8 m/s) may suggest stenosis or elevated downstream resistance (e.g., elevated PVR or branch PA stenosis). ° Values > 0.7–0.8 m/s raise concern for stenosis, obstruction, or collaterals.

## Data Availability

The data presented in this study is available on request from the corresponding author. The data is not publicly available due to privacy issues.

## Supplementary Materials

The following supporting information can be downloaded at: https://www.mdpi.com/article/10.3390/jcm15093520/s1, Supplemental Movie S1: Focused view on coronary artery showing coronary artery steal (red and blue flow) in HLHS after Norwood stage 1 with BTM shunt. Movie S1A: Subcostal color view of a restrictive (4.5 mm) foramen ovale in a neonate (2 days old, 3 kg of weight) with HLHS. Movie S1B: Subcostal color view of HLHS with restrictive PFO after atrial stenting. Movie S1C: Subcostal color view of HLHS with large ASD. Movie S2: Mildly decreased ventricular function in a 3-month-old, 4 kg female with hypoplastic left heart syndrome after initial Norwood 1 palliation with BTM shunt. Movie S3: Severe tricuspid regurgitation in a 1-month-old, 5 Kg male with HLHS after Norwood 1 palliation. Movie S3A: A 4-chamber view color Doppler showing severe tricuspid regurgitation. Movie S3B: 3D echocardiography showing a prolapse of tricuspid anterior leaflet. Movie S3C: Severe tricuspid regurgitation is imaged by blood speckle tracking echocardiography. Movie S4: A 12-year-old male with congenitally corrected transposition of the great arteries (CCTGA) after TCPC presenting with sub-aortic obstruction visualized in a modified 4-chamber view (Movie S4A); and a modified long-axis view (Movie S4B). Movie S5: Modified 4-chamber view of DKS, viewed with a novel BST imaging technique. Movie S6: Suprasternal view of a BTM shunt, viewed with a novel BST imaging technique. Movie S7: Modified 4-chamber view of a stenotic RV-PA Sano conduit. Movie S8: Parasternal view of a RV-PA Sano conduit visualized by a linear probe. Movie S9: Suprasternal view showing normal flow in Glenn conduit. Movie S10: (A) Subcostal view of normal flow from inferior vena cava to TCPC conduit; and (B) 4-chamber view of TCPC with flow across the fenestration. Movie S11: A 3-year-old with HLHS after Glenn operation with native aorta thrombosis visualized in long-axis view (A); modified 5-chamber view (B); and short-axis view (C). Movie S12: (A) A 4-chamber view, and (B) a short-axis view of a thrombus in LV in HLHS. Movie S13: Subcostal view of a thrombus in IVC in TCPC. Movie S14: Suprasternal view of a 5-year-old, 15 kg male immediately after Fontan operation with clots in the Glenn conduit. Movie S15: A modified 4-chamber view of a thrombus in TCPC conduct visualized in its extension. Movie S16: (A) Subcostal, and (B) 4-chamber view of a big clot external to the right section after TCPC. Table S1: Hypoplastic left heart syndrome (HLHS)/Severe aortic stenosis (AS)/borderline left ventricle/mitral atresia/aortic atresia. Table S2: Univentricular heart. Table S3: Post Norwood 1 (Sano vs BTM shunt). Table S4: Post bidirectional cavo-pulmonary connection (BCPC). Table S5: Post Total cavo-pulmonary connection (TCPC).
