# Supplementary material for: Systematic Echocardiographic Assessment of the Univentricular Heart Across the Stages of Fontan Palliation: A Practical Guide with Focus on Emerging 3D and 4D Imaging Modalities and Thromboembolic Complications from AEPC Imaging Working Group
Source: J Clin Med. 2026 May 5;15(9):3520. doi: 10.3390/jcm15093520 (PMC13165035; doi:10.3390/jcm15093520)
Supplement: Supplementary file 1 [file jcm-15-03520-s001.zip › jcm-4247224-supplementary materials.pdf]

Supplemental Movie S1 Focused view on coronary artery showing coronary artery steal (red and blue flow) in HLHS after Norwood stage 1 with BTM shunt.

Supplemental Tables

Supplemental Table S1: Hypoplastic left heart syndrome (HLHS) /Severe aortic stenosis (AS)/borderline left ventricle/mitral atresia/aortic atresia.

Patient Name
Age (years/months): Weight (kg): Length (cm): BSA (method):
Arterial Pressure (mmHg): Heart Rate: b.p.m. Oxygen Saturation:
Type of surgery: Surgery Date:
Echo machine: Software employed for 3D, strain analysis:
Department: Operator:

	Basic data	Advised Quantitative Measures	Adjunctive Measures and/or depending on associated defects
Situs_			
Position within the chest:			
Abdominal Aorta:			
Atrio-ventricular connection			
Ventriculo-arterial connection:			
Pulmonary venous return:			
Systemic venous return			
Inter-atrial septum	Bulging:<br>Size of the shunt:<br>Direction of the shunt:	V Max (m/s), Peak/mean gradient (mmHg)	
Inter-ventricular septum	Bulging:		
Cardiac Chamber			
Right atrium			
Left atrium			
Left ventricle:	Dimensions:<br>Cynesis:<br>Diastolic function:<br>Walls: Fibroelastosis:	M-mode<br>Biplane Volumes<br>LV AP diameter°<br>LAR°<br>EFE:	
Right ventricle	Dimensions:<br>Cynesis:<br>Diastolic function:<br>Walls:	RV lengths, areas, FAC, TAPSE, TD′ s′ lateral<br>AP diameter°	
Valves			
Tricuspid valve	Regurgitation:	Annulus (mm, Z-score)	

	Presence of moderate or severe TR°		
Mitral valve	Regurgitation:<br>Stenosis:<br>Papillary muscle:	V Max (m/s), Peak/mean gradient (mmHg)<br>PHT (ms)<br>Annulus: (mm, Z-score<br>MV Area (cm2)°<br>MVAi (cm2/m2)	
Aortic valve	Regurgitation:	V Max (m/s), Peak/mean gradient (mmHg)<br>Annulus : (mm, Z-score:), °<br>Root : (mm, Z-score:), °<br>Junction: (mm, Z-score:),<br>Asc Ao: (mm, Z-score), °<br>Sub Ao diameter: (mm, Z-score) °	
Pulmonary valve	Regurgitation: none, mild, moderate, severe	V Max (m/s), Peak/mean gradient (mmHg)	
Great vessels			
Main pulmonary artery:	Dimensions/Anatomy:	Diameters mm (Z-score)<br>V Max (m/s), Peak/mean gradient (mmHg)	
Right pulmonary artery:	Dimensions:<br>Anatomy:		*If Stenosis*<br>Diameter (mm, Z-score:)<br>V Max (m/s), Peak/mean gradient (mmHg)<br>Narrowest point (mm)
Left pulmonary artery:	Dimension/Anatomy:		*If Stenosis*<br>Diameter (mm, Z-score:)<br>V Max (m/s), Peak/mean gradient (mmHg)<br>Narrowest point (mm)
Aortic arch:	Dimensions:<br>Anatomy:	V Max (m/s), Peak/mean gradient (mmHg))<br>Run-off: Reverse flow:<br>*Diameters and z scores*	
Arterial duct/Collaterals			
Coronary arteries	Origin:<br>Dimensions:<br>Synosoid:		
Pericardium/ pleure	Effusion/Other		
Abdominal Aorta	Pulsatility		
Inferior vena cava/Heatic veins	Excursion<br>Congestion:<br>Reversal Flow:		
Quality of the examination:	Acoustic window:<br>Patient collaboration:		
Conclusions			

Z-score sources:

Ao=aorta; Abd Ao=abdominal aorta; Asc Ao=ascending aorta; BSA= body surface area: b.p.m.= beats per minute, Desc Ao=descending aorta; EFE=endocardial fibro-elastosis; IA=innominate artery, *LAR* LV long-axis to heart long-axis ratio,, LCA=left carotid artery; LSA=left subclavian artery; FAC= fractional area change; PA=pulmonary artery; *MVAi=* mitral valve area index PHT=pressure half time, RA=right atrium; RV=right ventricle; TAPSE=tricuspid annular plane systolic excursion, TD=I=tissue doppler imaging; 3D=three dimensional

Rodhes Score : 14.0 (BSA) + 0.943 (ROOTi) + 4.78 (LAR) + 0.157 (MVAi) − 12.03

Congenital Heart Surgeons' Society (CHSS) 1 Survival benefit = 30.55 (inverse of age at study entry [d] − 1) − 6.20 (aortic root *z*-score) − 12.14 (echocardiographic grade of EFE) − 23.33 (logarithm of Asc Ao [mm]) − 28.30 (presence of moderate or severe TR) − 0.70 (LV long-axis length *z*-score) − 86.47

CHSS2: Multivariable regression equation using BSA, MV *z*-score, indexed heart long axis, minimum LVOT diameter, mid aortic arch indexed.

Discriminating score 10.98 (BSA) − 0.56 (AoV *z*-score) − 5.89 (LAR) − 0.79 (presence of grade 2 or 3

EFE) − 6.78

Supplemental Table S2: Univentricular heart.

Patient Name
Age (years/months): Weight (kg): Length (cm): BSA (method):
Arterial Pressure (mmHg): Heart Rate: b.p.m. Oxygen Saturation:
Department: Operator:
Type of surgery: Surgery Date:
Echo machine: Software employed for 3D, strain analysis:

	Basic data	Advised Quantitative Measures	Adjunctive Measures and/or depending on associated defects
Situs			
Position within the chest:	Levocardia:<br>Mesocardia: Dextrocardia:		
Atrio-ventricular connection	Concordant:<br>Discordant:<br>Position of the aorta:<br>Position of the PA:		
Ventriculo-arterial connection:	Concordant:<br>Discordant:<br>Position of the aorta:<br>Position of the PA:		

Pulmonary venous return:	Normal:<br>Abnormal: right veins:<br>Left veins:		
Systemic venous return	Normal:<br>Abnormal:<br>IVC:<br>SVC:<br>LSVC:		
Inter-atrial septum	Bulging:<br>Size of the shunt:<br>Direction of the shunt:	V Max (m/s), Peak/mean gradient (mmHg))	
Inter-ventricular septum	Bulging:<br>Size of the shunt:<br>Direction of the shunt:		
Cardiac Chamber			
Right atrium			
Left atrium			
Left ventricle:	Dimensions:<br>Cynesis:<br>Diastolic function:<br>Walls:	M-mode_<br>Biplane Volumes	
Right ventricle	Dimensions:<br>Cynesis:<br>Walls:	RV lengths, areas, FAC, TAPSE, TD' s' lateral	
Valves			
Tricuspid valve	Regurgitation:	Estimated RV pressure: (mmHg)<br>Annulus: (mm, Z-score)	
Mitral valve	Regurgitation:	V Max (m/s), Peak/mean gradient (mmHg))<br>Annulus: (mm Z-score)	PHT (ms) mitral valve area (cm2)
Aortic valve	Regurgitation:	V Max (m/s), Peak/mean gradient (mmHg))<br>Diameters and z-scores	
Pulmonary valve	Regurgitation:	V Max (m/s), Peak/mean gradient (mmHg))<br>Annulus: (mm, Z-score)	
Great vessels			
Main pulmonary artery:	Dimensions:<br>Anatomy:	Diameter (mm, Z-score:)<br>V Max (m/s), Peak/mean gradient (mmHg)	
Right pulmonary artery:	Dimensions:<br>Anatomy:		<u>*If Stenosis*</u><br>Diameter (mm, Z-score:)<br>V Max (m/s), Peak/mean gradient (mmHg)<br>Narrowest point mm
Left pulmonary artery:	Dimensions:<br>Anatomy::		<u>*If Stenosis*</u><br>Diameter (mm, Z-score:)<br>V Max (m/s), Peak/mean gradient (mmHg)<br>Narrowest point mm

Aortic arch:	Dimensions:<br>Anatomy:<br>Anterograde Flow<br>Retrograde Flow:	V Max (m/s), Peak/mean gradient (mmHg)Run-off:<br>Reverse flow:<br>*Diameters and z scores*	
Arterial duct/Collaterals			
Coronary arteries	Origin:<br>Dimensions:		
Pericardium/ pleure	Effusion/Other		
Abdominal Aorta	Pulsatility		
Inferior vena cava/Heatic veins	Excursion<br>Congestion:<br>Reversal Flow:		
Quality of the examination:	Acoustic window:<br>Patient collaboration:		
Conclusions			
Z-score sources:			

Ao=aorta: Abd Ao=abdominal aorta; Asc Ao=ascending aorta; BSA= body surface area: b.p.m.= beats per minute, IA=innominate artery, Desc Ao=descending aorta; LCA=left carotid artery; LSA=left subclavian artery; FAC= fractional area change; PA=pulmonary artery; PHT=pressure half time, RA=right atrium; RV=right ventricle; TAPSE=tricuspid anDepartment: Operator:

nular plane systolic excursion, TD=I=tissue doppler imaging; 3D=three dimensional

Supplemental Table S3. Post Norwood 1 (Sano vs BTM shunt).

Patient Name
Age (years/months): Weight (kg): Length (cm): BSA (method):
Arterial Pressure (mmHg): Heart Rate: b.p.m. Oxygen Saturation:
Type of surgery: Surgery Date:
Echo machine: Software employed for 3D, strain analysis:
Department: Operator:

	Basic data	Advised Quantitative Measures	Adjunctive Measures and/or depending on associated defects
Pulmonary venous return			
Systemic venous return	Haepatic congestion		
Inter-atrial septum	Bulging:<br>Size of the shunt:<br>Direction of the shunt:	V Max (m/s), Peak/mean gradient (mmHg))	
Inter-ventricular septum	Size of the shunt:<br>Direction of the shunt:	V Max (m/s), Peak/mean gradient (mmHg))	
Cardiac Chamber			
Right atrium			
Left atrium			
Systemic	Dimensions:<br>Cynesis:	M-mode:<br>Biplane Volumes:	

	Diastolic function:<br>Walls:	RV lengths, areas, FAC, TAPSE,<br>TD' s' lateral	
Accessory ventricle			
AV Valves			
Right sided valve	Regurgitation:	*Power and Tissue Doppler diastolic parameters*	
Left sided valve	Regurgitation:	*Power and Tissue Doppler diastolic parameters*	
Neo- Aortic valve	Regurgitation:	V Max (m/s), Peak/mean gradient (mmHg)	
Pulmonary valve	Regurgitation:	V Max (m/s), Peak/mean gradient (mmHg)	
Great vessels			
Main pulmonary artery:	Dimensions:<br>Anatomy:	Diameter (mm, Z-score:)<br>V Max (m/s), Peak/mean gradient (mmHg)	
Right pulmonary artery:	Dimensions:<br>Anatomy:		*If Stenosis*<br>Diameter (mm, Z-score:)<br>V Max (m/s), Peak/mean gradient (mmHg)Narrowest point mm
Left pulmonary artery:	Dimensions:<br>Anatomy:		*If Stenosis*<br>Diameter (mm, Z-score:)<br>V Max (m/s), Peak/mean gradient (mmHg)Narrowest point mm
Aortic arch:	Dimensions:<br>Anatomy:<br>Anterograde flow:<br>Retrograde Flow:	V max (m/s)	*If Stenosis*<br>V Max (m/s), Peak/mean gradient (mmHg)Run-off:<br>Reverse flow:<br>*Diameters and z-scores*
Arterial duct/Collaterals/Shunt/Conduit			
Coronary arteries	Origin:<br>Dimensions:<br>Flow:		
Pericardium/ pleure	Effusion/Other		Effusion max diameter (mm)
Abdominal Aorta	Pulsatility		V max (m/s)<br>Dec time (ms)<br>Acc Time (ms)
Inferior vena cava/Heatic veins	Excursion<br>Congestion:<br>Reversal Flow:		Systolic Diameter mm<br>Diastolic Diameters (mm)
Clots/Thrombi			
Quality of the examination:	Acoustic window:<br>Patient collaboration:		
Conclusions			

Z-score sources:

Ao=aorta, Abd Ao= abdominal aorta; Acc=acceleration, Dec=deceleration: BSA= body surface area: b.p.m.= beats per minute, FAC= fractional area change; PA=pulmonary artery; RV=right ventricle; TAPSE=tricuspid annular plane systolic excursion, TD=I=tissue doppler imaging; 3D=three dimensional

Supplemental Table S4. Post bidirectional cavo-pulmonary connection (BCPC).

Patient Name
Age (years/months): Weight (kg): Length (cm): BSA (method):
Arterial Pressure (mmHg): Heart Rate: b.p.m. Oxygen Saturation:
Department: Operator:
Type of surgery: Surgery Date:
Echo machine: Software employed for 3D, strain analysis:

	Basic data	Advised Quantitative Measures	Adjunctive Measures and/or depending on associated defects
Pulmonary venous return			
Systemic venous return	Haepatic congestion		
Inter-atrial septum	Bulging:<br>Size of the shunt:<br>Direction of the shunt:	V Max (m/s), Peak/mean gradient (mmHg))	
Inter-ventricular septum	Size of the shunt:<br>Direction of the shunt:	V Max (m/s), Peak/mean gradient (mmHg))	
Cardiac Chamber			
Right atrium			
Left atrium			
Systemic	Dimensions:<br>Cynesis:<br>Diastolic function:<br>Walls:	M-mode_<br>Biplane Volumes<br>RV lengths, areas, FAC, TAPSE, TD’ s’ lateral	
Accessory ventricle			
AV Valves			
Right sided valve	Regurgitation:	*Power and Tissue Doppler diastolic parameters*	
Left sided valve	Regurgitation:	*Power and Tissue Doppler diastolic parameters*	
Neo- Aortic valve	Regurgitation:	Max velocity: cm/s Mean velocity: cm/s	
Pulmonary valve	Anterogarde flow:<br>Regurgitation:	Max velocity: cm/s Mean velocity: cm/s	
Great vessels			
Main pulmonary artery:	Dimensions/Anatomy:	Diameter (mm, Z-score:)<br>V Max (m/s), Peak/mean gradient (mmHg)	
Right pulmonary artery:	Dimensions/Anatomy:		*<u>If Stenosis</u>*<br>Diameter (mm, Z-score:)<br>V Max (m/s), Peak/mean gradient (mmHg))<br>Narrowest point mm

Left pulmonary artery:	Dimensions/Anatomy:		*If Stenosis*<br>Diameter (mm, Z-score:)<br>V Max (m/s), Peak/mean gradient (mmHg)Narrowest point mm
Aortic arch:	Dimensions/Anatomy:<br>Anetrograde flow:<br>Retrograde Flow:	V max (m/s)	*If Stenosis*<br>V Max (m/s), Peak/mean gradient (mmHg)Run-off:<br>Reverse flow:<br>*Diameters and z-scores*
Cavo-pulmonary connection:	Caliber:<br>Flow:		
Coronary arteries	Origin:<br>Dimension:<br>Flow:		
Pericardium/ pleure	Effusion/Other		Effusion max diameter (mm)
Abdominal Aorta	Pulsatility		V max (m/s), Dec time (ms), Acc Time (ms)
Inferior vena cava/Heatic veins	Excursion<br>Congestion:<br>Reversal Flow:		Systolic Diameter (mm)<br>Diastolic Diameters (mm)
Clots/Thrombi			
Quality of the examination:	Acoustic window:<br>Patient collaboration:		
Conclusions			
Z-score sources:			

Ao=aorta, Abd Ao=abdominal Aorta: Asc Ao=ascending aorta, Desc Ao=descending aorta; Acc=acceleration, Dec=deceleration; BSA= body surface area: b.p.m.= beats for minute, FAC= fractional area change; PA=pulmonary artery; RV=right ventricle; TAPSE=tricuspid annular plane systolic excursion, TD=I=tissue doppler imaging; 3D=three dimensional

Supplemental Table S5: Post Total cavo-pulmonary connection (TCPC).

Patient Name
Age (years/months): Weight (kg): Length (cm): BSA (method):
Arterial Pressure (mmHg): Heart Rate: b.p.m. Oxygen Saturation:
Type of surgery: Surgery Date:
Echo machine: Software employed for 3D, strain analysis:
Department: Operator:

	Basic data	Advised Quantitative Measures	Adjunctive Measures and/or depending on associated defects
Pulmonary venous return			
Systemic venous return	Haepatic congestion		
Inter-atrial septum	Bulging:<br>Size of the shunt:<br>Direction of the shunt:	V Max (m/s), Peak/mean gradient (mmHg))	

Inter-ventricular septum	Size of the shunt:<br>Direction of the shunt:	V Max (m/s), Peak/mean gradient (mmHg))	
Cardiac Chamber			
Right atrium			
Left atrium			
Systemic	Dimensions:<br>Cynesis:<br>Diastolic function:<br>Walls:	M-mode:<br>Biplane Volumes:<br>RV lengths, areas, FAC, TAPSE, TD’ s’ lateral	
Accessory ventricle			
AV Valves			
Right sided valve	Regurgitation:	Power and Tissue Doppler diastolic parameters	
Left sided valve	Regurgitation:	Power and Tissue Doppler diastolic parameters	
Neo- Aortic valve	Regurgitation:	V Max (m/s), Peak/mean gradient (mmHg)	
Pulmonary recess			
Great vessels			
Right pulmonary artery:	Dimensions/Anatomy:	Diameter (mm, Z-score:)<br>V Max (m/s), Peak/mean gradient (mmHg)<br>Narrowest point mm	
Left pulmonary artery:	Dimensions/Anatomy:	V max m/s, Max/mean gradient mmHg<br>Diameters mm (Z-score)<br>Narrowest point mm	
Aortic arch:	Dimensions/Anatomy:<br>Anterograde flow:<br>Retrograde Flow:	V Max (m/s), Peak/mean gradient (mmHg)<br>Run-off: Reverse flow:	*Diameters and z-scores*
Superior Cavo-pulmonary connection:	Caliber<br>Flow:	Size:<br>Size at the level of the anastomosis	
Inferior Cavo-pulmonary connection:	Caliber:<br>Flow:<br>Fenestration.	Size:<br>Size at the level of the anastomosis<br>Fenestration gradient max/mean (mmHg)	
Coronary arteries	Origin:<br>Dimensions:<br>Flow:		:
Pericardium/ pleure	Effusion/Other		Effusion max diameter (mm)
Abdominal Aorta	Pulsatility		V max (m/s), Dec time (ms), Acc Time (ms)
Inferior vena cava/Heatic veins	Excursion<br>Congestion:<br>Reversal Flow:		Systolic Diameter (mm)<br>Diastolic Diameters (mm)
Clots/Thrombi			
Quality of the examination:	Acoustic window:<br>Patient collaboration:		

Conclusions:

Ao=aorta, Desc Ao=descending aorta; Acc=acceleration; Dec=decelerationBSA= body surface area: b.p.m.= beats per minute, FAC= fractional area change; PA=pulmonary artery; RV=right ventricle; TAPSE=tricuspid annular plane systolic excursion, TD=I=tissue doppler imaging; 3D=three dimensional.
